# Adaptive Multi-Factor Quantitative Analysis and Prediction Models: Vaccination, Virus Mutation and Social Isolation on COVID-19

**DOI:** 10.3389/fmed.2022.828691

**Published:** 2022-03-16

**Authors:** Yuanyuan Pei, Juan Li, Songhua Xu, Yi Xu

**Affiliations:** ^1^Clinical Data Center, Guangzhou Women and Children's Medical Center, Guangzhou Medical University, Guangzhou, China; ^2^Department of Infectious Diseases, Guangzhou Women and Children's Medical Center, Guangzhou Medical University, Guangzhou, China

**Keywords:** adaptive multi-factor model, COVID-19, vaccination, virus mutation, social isolation

## Abstract

Different countries have adopted various control measures for the COVID-19 pandemic in different periods, and as the virus continues to mutate, the progression of the pandemic and preventive measures adopted have varied dynamically over time. Thus, quantitative analysis of the dynamic impact of different factors such as vaccination, mutant virus, social isolation, etc., on transmission and predicting pandemic progress has become a difficult task. To overcome the challenges above and enable governments to formulate reasonable countermeasures against the ongoing COVID-19 pandemic, we integrate several mathematical methods and propose a new adaptive multifactorial and geographically diverse epidemiological model based on a modified version of the classical susceptible-exposed-infectious-recovered (SEIR) model. Based on public datasets, a multi-center study was carried out considering 21 regions. First, a retrospective study was conducted to predict the number of infections over the next 30 days in 13 representative pandemic areas worldwide with an accuracy of 87.53%, confirming the robustness of the proposed model. Second, the impact of three scenarios on COVID-19 was quantified based on the scalability of the model: two different vaccination regimens were analyzed, and it was found that the number of infections would progressively decrease over time after vaccination; variant virus caused a 301.55% increase in infections in the United Kingdom; and 3-tier social lockdown in the United Kingdom reduced the infections by 47.01%. Third, we made short-term prospective predictions for the next 15 and 30 days for six countries with severe COVID-19 transmission and the predicted trend is accurate. This study is expected to inform public health responses. Code and data are publicly available at https://github.com/yuanyuanpei7/covid-19.

## Introduction

With the global spread of the coronavirus disease-2019 (COVID-19) to over 200 countries (1), different countries or regions have taken various measures to curb the pandemic, such as wearing masks, maintaining social distancing, contact tracing, regional blockades, etc. (2, 3); many countries are also actively implementing vaccination plans (4, 5). However, the effectiveness of available vaccines and the timelines for implementing vaccination vary between countries, and some countries are still facing vaccine shortages (6). Furthermore, the virus mutates, leading to variants such as B.1.1.7 and B.1.351 (7, 8). In addition, the control measures applied have also varied throughout the pandemic in different stages. As a result of the factors above, quantitative analysis and prediction of the spread of the COVID-19 pandemic are challenging.

Mathematical modeling is an effective method for quantitative analysis of the COVID-19 pandemic. There are two main types of these models, the propagation dynamics models represented by SEIR and the probability models. Their functions are: analyzing the dynamic transmission of COVID-19, assessing the impact of different factors on the COVID-19 pandemic, and predicting the pandemic trend. Through mathematical modeling, it is helpful for early intervention, reasonable allocation of medical resources, and helping the government to formulate response measures. For example, Della Rossa et al. demonstrated an analysis model indicating that intermittent regional strategies can alleviate the pandemic (9). Gu et al. used a predictive model to assess the number of deaths between the apex and endpoints of the COVID-19 pandemic (10). Reno et al. used the SEIR model to predict the dynamic spread of COVID-19 and its burden on hospital care under different social distances (11). Kissler et al. used an improved SEIR model to predict the COVID-19 spread and emphasized the importance of intermittent distancing and serum monitoring (12). Russell et al. studied the effect of internationally imported cases on domestic transmission (13). Bayham and Fenichel analyzed the impact of closing schools on American healthcare workers during the pandemic (14). Li et al. used age-structured modifications of the SEIR model to investigate how to reduce the spread of COVID-19 (15). All these models have contributed to the study of the COVID-19 pandemic. The SEIR model shows good performance for the dynamic transmission of SARS-CoV-2 in different populations but requires a large number of cases to simulate, while the probability model has the advantage of analyzing the data but does not depict the dynamic propagation process well. However, these models were studied early in the COVID-19 pandemic when there was not enough knowledge about COVID-19, for example, Della Rossa et al. did not predict the number of cases and deaths (9). Moreover, no vaccines were used and no new mutant viruses emerged in the early years of the pandemic. Hence, it is vital to study multifactorial epidemiological models applicable to different geographical regions in the new pandemic environment.

The pandemic, populations, and various restraining measures interact with one another and vary over time (16), consistent with the descriptions of the susceptible-exposed-infectious-recovered (SEIR) model regarding the transmission dynamics of infectious diseases. SEIR is a compartmental model that is often applied to the mathematical modeling of infectious diseases, wherein the population is assigned to compartments with labels, for example, S, E, I, or R (Susceptible, Exposed, Infectious, or Recovered), and people progress among compartments. In this study, we propose a new approach designed to perform a quantitative analysis of the impact of various interventions on the pandemic by modifying the classical SEIR propagation dynamics model and combining it with ordinary differential equations and mathematical integration functions. We selected data from 21 regions distributed in different parts of the world that are the most significant contributors to the outbreak and performed seven experiments to quantitatively analyze the impact of two vaccination methods, viral variants, and social blockade on the spread of the pandemic, additionally, the progress of the pandemic is predicted.

## Methods

### Data Sets

In this multi-center study including data on 21 countries or regions, sourced from the Johns Hopkins Center for Systems Science and Engineering (JHU.CSSE) and the World Bank's public dataset for Global Health, and OpenStreetMap (1, 17), the collected publicly available datasets includes newly diagnosed infections per day, existing confirmed infections, number of people recovered, and deaths. Globally, we selected some of the countries and regions most affected by the pandemic based on infection rates and numbers of infections, and then excluded some countries and regions by checking the completeness of the raw data. In addition, Considering the COVID-19 distribution in different regions, for example, the pandemic in Hong Kong is not particularly severe worldwide, but it is severe in East Asia. Finally, we identified 21 countries and regions.

We also collected other publicly available data from the Internet, including the total population data of the 21 countries or regions mentioned above. To study the impact of different vaccination methods on the pandemic, we chose New York, owing to New York's easily available data and large population, we also obtained its daily vaccination data by consulting publicly available government information, and it was confirmed that the majority of people were administered the Pfizer vaccine (18). Furthermore, the effectiveness of the Pfizer vaccine has been reported in the literature (19, 20). In addition, we obtained the date of the initial discovery and spread of the UK virus variant (21) as well as the start date of the UK 3-tier lockdown (22). All of the information above was used in this study. We have published the data and code used to model the 21 regions mentioned here experimentally; it is available at the URL https://github.com/yuanyuanpei7/covid-19.

As all the data of patients were de-identified, the requirement for written informed consent and ethical approval was waived. All procedures performed in studies involving human participants were in accordance with the National Research Committee and the 1964 Helsinki declaration and its later amendments or comparable ethical standards.

### Establishing a New Adaptive Multi-Factor Model

In this study, considering that the propagation of COVID-19 is determined by various factors, the classic SEIR model is modified (23). First, redefining the function of the model, (1) we consider the viral incubation period. (2) Patients in the incubation period are also infectious, however, they are less infectious than fully infected people. (3) Vaccination or social isolation of susceptible populations, the immune efficacy of the vaccine or the protective of the isolated population is between 0 and 1. Second, new dynamic propagation parameters “α” and “σ” are added to include the analysis functions of vaccination and social isolation in the model. Third, a set of ordinary differential equations has been developed based on the functions and transmission dynamics of the new model.

The population is classified according to the transmission dynamics of the new SEIR model, as follows.

S (Susceptible): A healthy person who lacks immunity is susceptible to infection after contact with an infected person.
E (Exposed): People who have been in contact with an infected person.
I (Infectious): Infectious patients can spread the virus to S and turn them into E or I.
R (Recovered): People who have immunity after recovery will not revert to S, E, or I.
N: The total number of people in an area, not considering new births and immigration and deaths. This total number remains unchanged.

The definition of parameters is crucial to realizing the functions of the model. According to literature reports, we set or adjust the values of these parameters in the model (24–27). Different geographical areas have different epidemiological characteristics, such as diverse populations and densities, interventions, and viral variations, and therefore, the values of some parameters are variable, such as “β”, “*q*,” “α”, “σ”, and “t”. Other parameters have fixed values, such as “ε” being the reciprocal of the incubation period (5.2 days) and “γ” being the reciprocal of the recovery time of the infections (14 days). In the new model, the parameters are defined as follows.

β: Infection rate of the infections. β means: for example, that on average, an infected person is exposed to M individuals and the probability of infection after exposure is P (0~1), β = M^*^P, Therefore, β > 0.
*q*: The infectivity of latent relative to infections, with a ratio between 0 ~1.
α: The vaccination rate of the susceptible population and the isolation rate of the susceptible population. For example: 20 out of 100 people are vaccinated, and the other ten people are quarantined, the value of α is 30%. Therefore, the value of α is 0 ~ 1.
σ: The protective effect of the vaccine and the effectiveness of isolation. For example, 50 out of 100 people are vaccinated and 30 develop an immune response. The other 50 are isolated and 30 of them are fully protected, so the value of q is 60%. Therefore, the value of σ is 0 ~ 1.
ε: Proportion of latent converted to infections, reciprocal of 5.2 days.
γ: Proportion of persons recovered from infections, reciprocal of 14 days.
*t*: Represents the number of days.

In the new model, the population of each category is dynamically changed, which is expressed by ordinary differential mathematical equations as follows.

Rate of transmission from infections to susceptible (unvaccinated or unisolated susceptible) is obtained as


(1) $$\begin{array}{r} \beta S\left(1-\alpha \right)I/N \end{array}$$

Rate of transmission from latent to susceptible (unvaccinated or unisolated susceptible persons) is obtained as


(2) $$\begin{array}{r} \beta S\left(1-\alpha \right)qE/N \end{array}$$

Rate of transmission from infections to susceptible (people who have been vaccinated but have not acquired immunity or people who have been infected because of insufficient quarantine measures) is obtained as


(3) $$\begin{array}{r} \beta \alpha S\left(1-\sigma \right)I/N \end{array}$$

Rate of transmission from latent to susceptible (vaccinated but unimmunized) is obtained as


(4) $$\begin{array}{r} \beta \alpha S\left(1-\sigma \right)qE/N \end{array}$$

The new SEIR model considers that latent patients are also infectious, but does not account for new immigrant populations, neonatal populations, or natural deaths. The new SEIR population expression formula is as follows:


(5) $$\begin{array}{r} N=S+E+I+R \end{array}$$

In the new SEIR model, a set of ordinary differential equations represents the dynamic spread of the four groups of “susceptibility,” “exposure,” “infection,” and “recovery” as follows.


(6) $$\begin{array}{r} dS/dt=-\beta S\left(1-\alpha \right)I/N-\beta S\left(1-\alpha \right)qE/N-\beta \alpha S\left(1-\sigma \right)I/N\\ \;-\beta \alpha S\left(1-\sigma \right)qE/N-\sigma \alpha S \end{array}$$

(7) $$\begin{array}{r} dE/dt=\beta S\left(1-\alpha \right)I/N+\beta S\left(1-\alpha \right)qE/N+\beta \alpha S\left(1-\sigma \right)I/N\\ \;+\beta \alpha S\left(1-\sigma \right)qE/N-\varepsilon E \end{array}$$

(8) $$\begin{array}{r} dI/dt=\varepsilon E-\gamma I \end{array}$$

(9) $$\begin{array}{r} dR/dt=\sigma \alpha S+\gamma I \end{array}$$

It is worth noting that formula (6) represents the constant removal rate from the susceptible population. The susceptible people have been dynamically decreasing during the pandemic spread, and it will only decrease. Therefore, the first four rates of formula (6) are <0. σα*S* indicates the rate of increase in the number of people gaining protection after being vaccinated and being isolated among susceptible individuals. The actual rate of being protected is also the rate of moving out of the susceptible population daily, so the rate is also <0.

In formula (9), γI represents the rate of the population recovering from the infected each day. σα*S* (Rate of increase in the number of gaining protection through vaccination or isolation in susceptible individuals) is constantly moving out of the susceptible population to the recovered population in the form of a negative number. It is increasing in the recovered population, so σα*S* is a positive number in formula (9). The recovered population is divided into two parts, those who have recovered from the infections and those who have been protected through vaccination and isolation, and therefore are the sum of the above two parts.

Based on the explanation of formulas (6) and (9), formulas (7) and (8) can also be understood, and relevant references are attached (23, 28).

### Functions and Scalability

**Figure 1:**
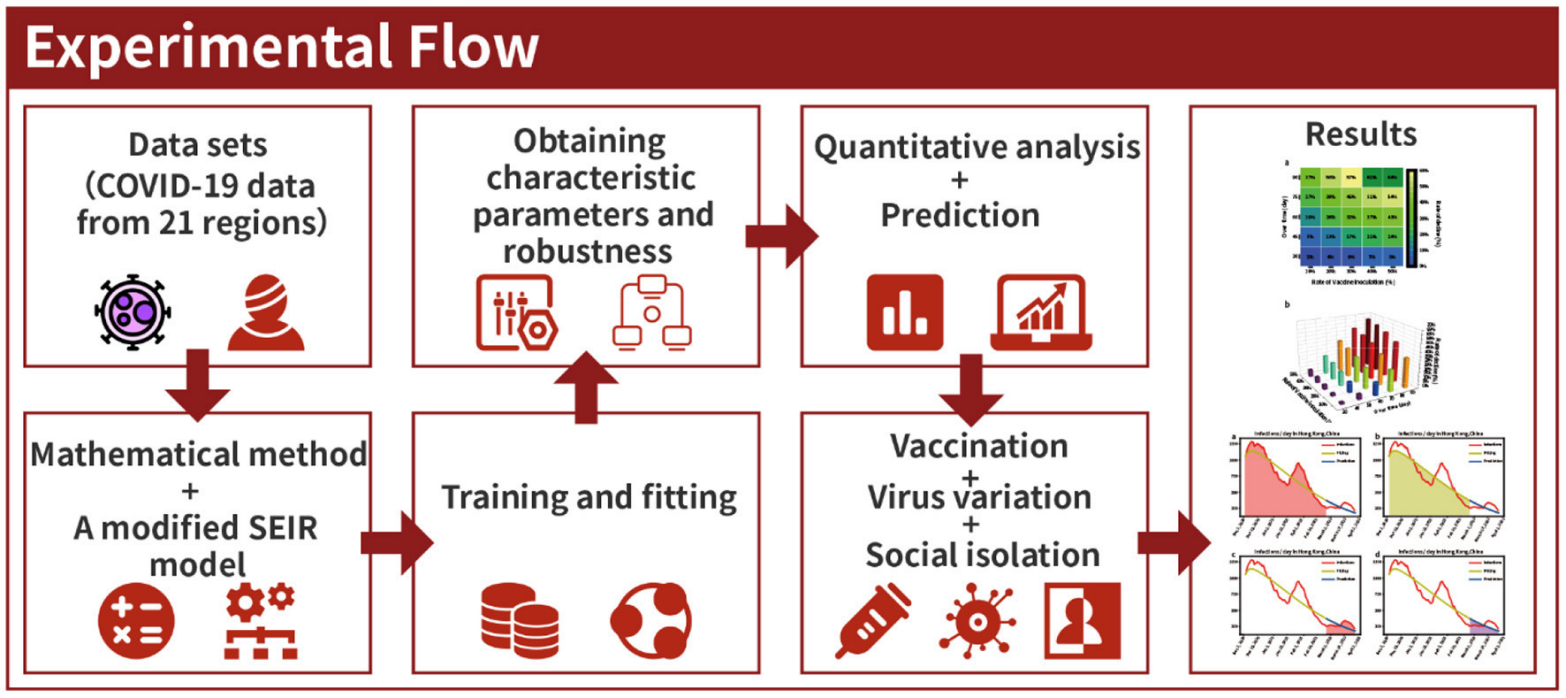
Experimental flow.

The experimental flow is shown in Figure 1.

These procedures were performed according to the following steps.

Step 1: The model is defined by the function “SEIR_model.” Loading the daily data table “pd.read_csv” of existing infected people and the area's population into the “SEIR_model” function of the model. The number of recovered people was loaded directly onto the program.
Step 2: Ensuring the model's adaptability through training and fitting historical data is the focus of this study. Owing to different characteristics of the pandemic in different regions and different stages, appropriate upper and lower limits were set for the parameter group (β, *q*, α, σ, ε, and γ). Then the historical data was trained and fitted 20 million times through the “optimize.curve_fit” function. Finally, a set of optimal parameters and best-fitting curves are obtained automatically.
Step 3: After loading different factors of the model (vaccination, viral mutation, social distancing, and the prediction of pandemic), new quantitative analysis functions are achieved by calculating the area under the curve using a mathematical integral equation, given as follows.
(10) $$\begin{array}{r} y=\int_{start}^{end}I\left(t\right)dt \end{array}$$

The formula (10) represents the total time of the disease course of all people in the infection period during the START and END periods. The area under the integral curve represents the entire disease course of the infections during that period, which helps to clearly describe the pandemic's impact on the population and the economy.

### Experimental Environment

All the experiments were performed on a personal computer (Windows 7 Home Edition, 64-bit operating system), the installed software was PyCharm Professional 2020.

## Results

Seven studies were carried out, the first six of which were retrospective and the last prospective research, yielding the following results.

### Prediction of the Pandemic in 13 Regions

Through training and fitting actual historical data, the COVID-19 trend curves can be obtained and used to predict the pandemic trend (28–30). According to this principle, in 13 regions with severe pandemic transmission distributed in different locations worldwide, historical data were used to train and fit the new model, and the number of infections in the next 30 days was predicted. The two phases of the experiment were as follows.

**Figure 2:**
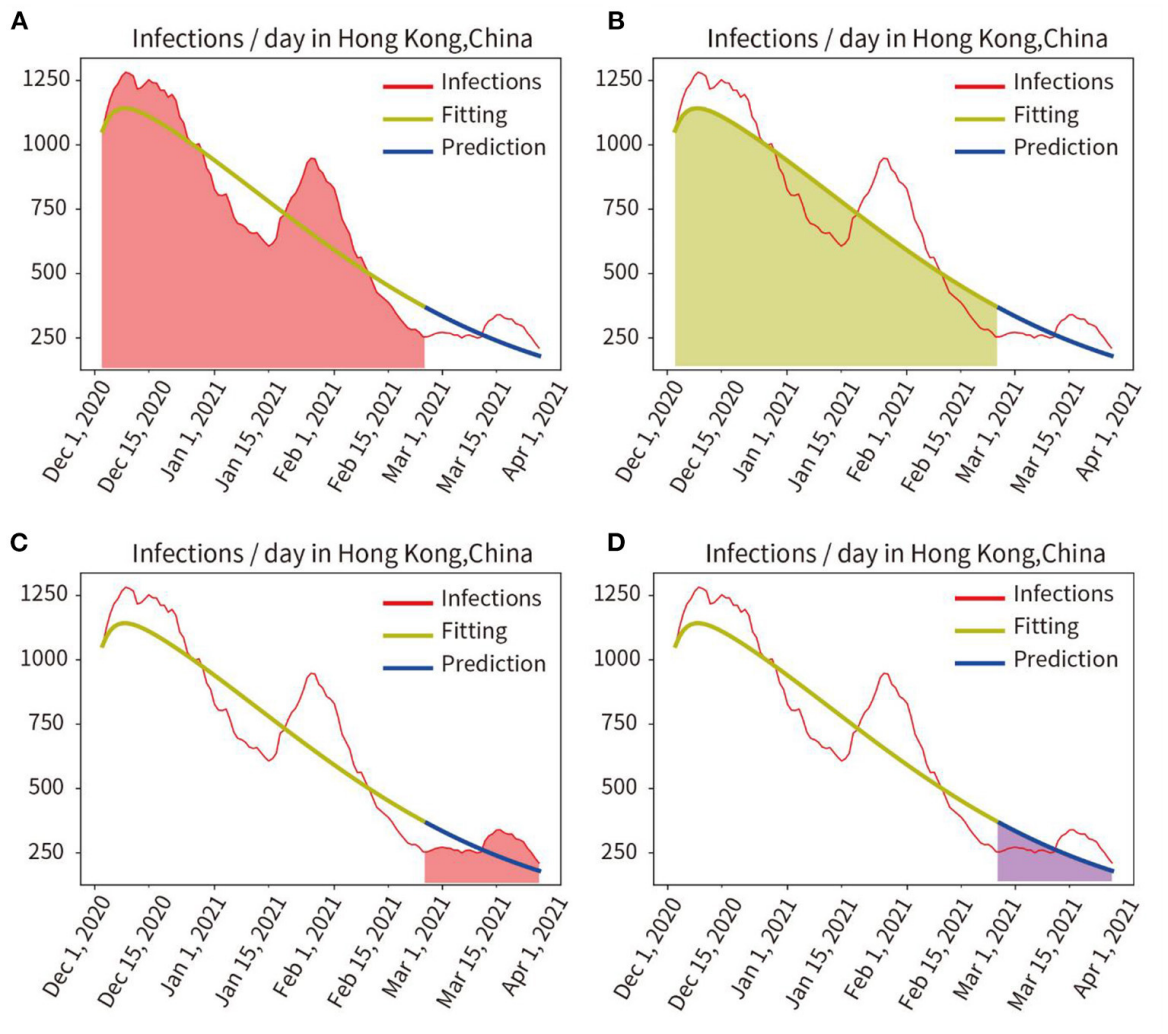
Quantitative analysis of fitting and prediction for HongKong. **(A)** Red area represents the integral under the curve of actual infections during the fitting period. **(B)** Yellow area represents the integral under the curve of the fitted infections during the fitting period. **(C)** Red area indicates the integral under the curve of actual infections in the prediction period. **(D)** Blue area represents the integral under the curve of predicted infections in the prediction period.

Phase I: The red line in Figure 2 represents the number of actual infections per day. First, the model was trained, and the actual pandemic data of Hong Kong from 31 December, 2020 to 24 February, 2021 were fitted, the fitted curve was obtained as the yellow line in Figure 2. Parameters of the model adapted to the pandemic characteristics during this time were also obtained. Then, using the parameters obtained by fitting and training, the model predicted the number of infected persons between 25 February and 26 March 2021, as shown in the blue curve in Figure 2.

The actual and fitted values were obtained by calculating the mathematical integration of the red area in Figure 2A and the yellow shaded area in Figure 2B. And the accuracy of the fit was obtained as 97.24% (fitted/actual). We also compared the integration of the red curve (actual number of infections) with the blue curve (predicted number of infections) for the period from 25 February to 26 March, 2021, as shown in Figure 2C red area and Figure 2D blue area, and obtained a prediction accuracy of 95.29%.

**Figure 3:**
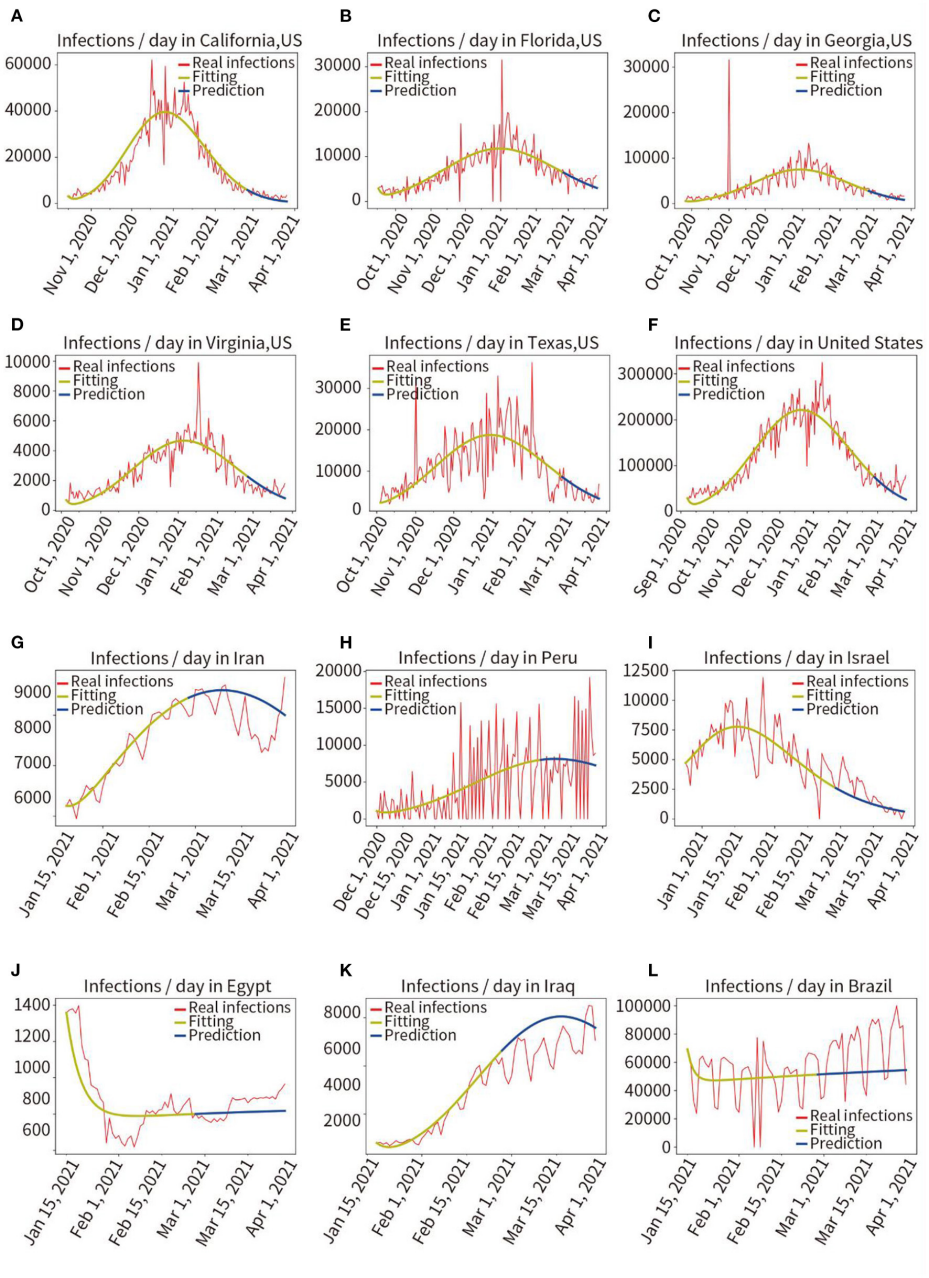
Retrospective quantitative prediction for 12 countries or regions. Actual infected individuals are represented by red curves. Fitted infections are represented by yellow curves. Predicted infections are represented by blue curves. **(A)** California, **(B)** Florida, **(C)** Georgia, **(D)** Virginia, **(E)** Texas, **(F)** the United States, **(G)** Iran, **(H)** Peru, **(I)** Israel, **(J)** Egypt, **(K)** Iraq, **(L)** Brazil.

Phase II: The proposed model was tested in 13 different countries and regions to verify its robustness. We selected eight countries worldwide and five states with a larger population and a more severe pandemic in the United States. As may be observed in Figure 3 and Table 1, the average fitting accuracy of the SEIR model for actual infections was 97.91%, and the prediction accuracy for the overall infected population during the following 30 days was 87.53%, which demonstrates the fitting and prediction accuracy, adaptability, and robustness of the model in different regions.

**Table 1:** Fitting and prediction accuracy for 13 countries and regions.

**Results of training and fitting using the pandemic data before the predicted start date**				**Results of retrospective prediction for the next 30 days**		
**Area**	**Real integral**	**Fitting integral**	**Fitting accuracy %**	**real integral**	**Prediction integral**	**Prediction accuracy %**
California	2,680,419	2,784,677	96.26	107,520	79,861	74.28
Florida	1,217,351	1,241,724	98.04	147,518	138,462	93.86
Georgia	686,137	677,325	98.72	55,516	50,718	91.36
Virginia	417,076	413,676	99.18	43,116	43,847	98.33
Texas	1,831,788	1,796,822	98.09	151,518	172,968	87.60
United States	22,543,471	22,541,901	97.06	1,878,409	1,438,641	72.45
Iran	266,868	277,238	96.26	240,490	250,904	95.85
Peru	353,923	356,882	99.17	213,540	237,144	90.05
Israel	381,102	378,118	99.76	61,144	42,749	81.56
Egypt	26,899	26,373	98.04	18,724	18,147	96.92
Iraq	76,130	79,508	95.75	142,795	170,067	83.96
Brazil	2,131,338	2,146,708	99.28	2,079,058	1,589,472	76.45
Hong Kong	68,271	66,389	97.24	8,359	7,965	95.29

### Effect of Vaccination on the Reduction of Infections

In New York, the initial COVID-19 vaccination drive was implemented from 3 December 2020 to 26 March 2021, in these 100 days, ~30% of the population was vaccinated at least once (18). The reference basis for the effectiveness of vaccination is as follows (19, 20): (1) The vaccine efficacy after the first shot was 56%. (2) The overall vaccine efficacy after the second shot was ~90%. (3) An immune response produced occurs in the body about 15 days after vaccination; therefore, we set the date when the vaccine became effective as 16 December 2020, and assumed that the population was inoculated at the same rate each day for the next 100 days, meaning the average daily vaccination rate was 0.3%.

**Figure 4:**
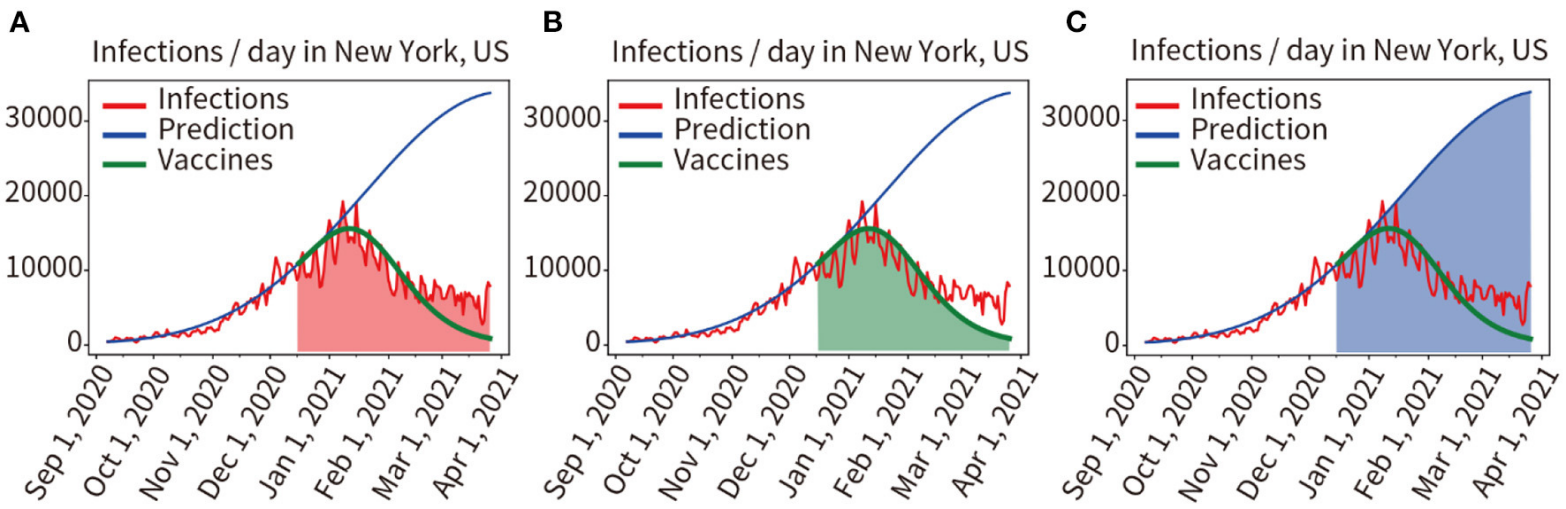
Quantitative analysis of the two scenarios of vaccinated and unvaccinated individuals. **(A)** Red area indicates the integral under the curve of actual infected individuals in the prediction period. **(B)** The green area indicates the integral under the predicted infected person curve in the vaccinated condition. **(C)** The blue area indicates the integral under the predicted infected person curve in the unvaccinated condition.

In Figure 4, the red curve indicated the actual number of infections per day. We used the model to train and fit pandemic data from 7 September, 2020 to 15 December, 2020 (vaccination on 3 December, 2020, immune response *in vivo* on 16 December, 2020), the obtained model and parameters were used to predict pandemic progression under unvaccinated conditions within 100 days after December 16, 2020, as shown in the blue curve in Figure 4C. Then, we used the model to predict pandemic progression in these 100 days under loaded vaccine conditions, the green prediction curve of vaccination in Figure 4A (the number of infections predicted per day after vaccination) is obtained.

Comparing the mathematical integral of the red curve for the actual number of infections (Red area in Figure 4A) with the mathematical integral of the green predicted curve for implementing the vaccination plan described above (Green area Figure 4B), the result was 916,966/970,234 = 94.51%. The prediction results of the model for loading vaccination conditions were confirmed to be accurate. We also compared the mathematical integral of the blue prediction curve assuming no vaccination (Blue area in Figure 4C) with that of the red curve for the actual number of infections (Red area in Figure 4A), yielding a result was 2,389,790:970,234. As shown in Figures 4A,C, it was found that vaccination reduced the number of infections by 59.40%.

### Impact of Different Vaccination Rates in the Population on the Pandemic

**Figure 5:**
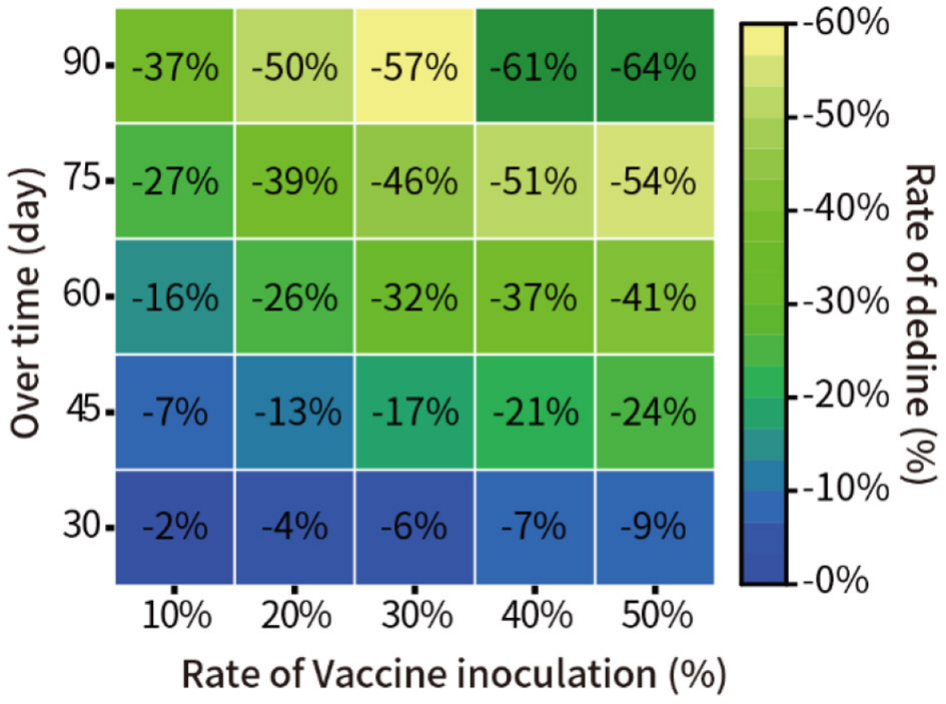
Prediction of the pandemic under different vaccination rates in New York.

Here, it was assumed that the vaccine efficacy was 90%, and the vaccination rate was 10, 20, 30, 40, and 50% of the total population of New York. The model was used to quantitatively predict the number of infections in the next 30, 45, 60, 75, and 90 days. The result of the relative decline of infections is shown in Figure 5.

### Impact of Vaccination With Different Potency on the Pandemic

**Figure 6:**
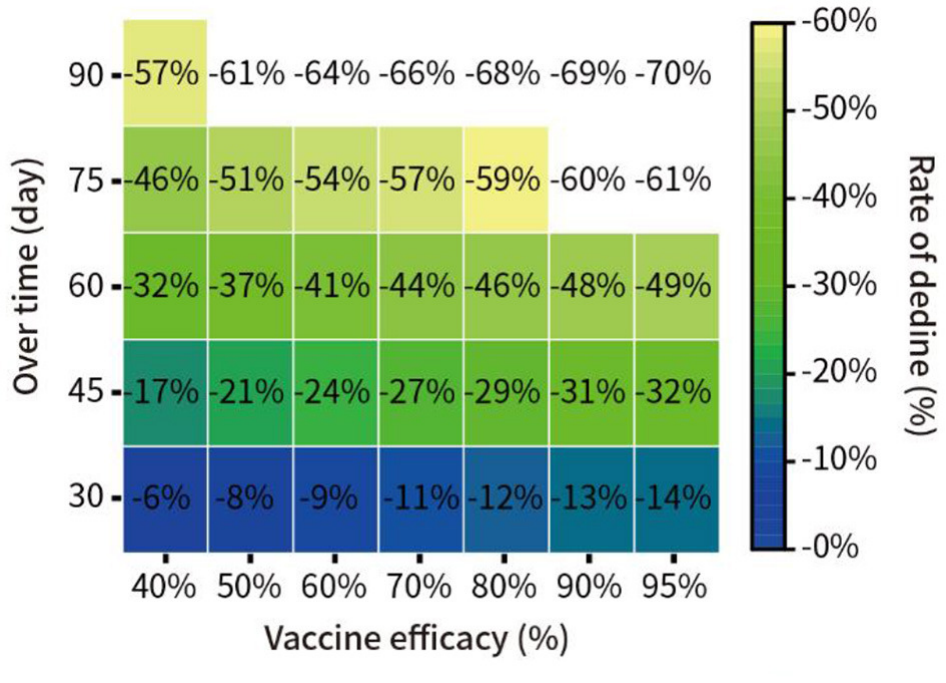
Prediction of the spread of the pandemic under vaccination with different potency in New York.

In addition, we assumed that 30% of the total New York population was vaccinated, with vaccine efficacy of 40, 50, 60, 70, 80, and 95%. We quantitatively predicted the decline in the proportion of infections at 30, 45, 60, 75, and 90 days from 16 December 2020. The results are shown in Figure 6.

### Quantitative Analysis of the Impact of Virus Variation on the Pandemic in the UK

**Figure 7:**
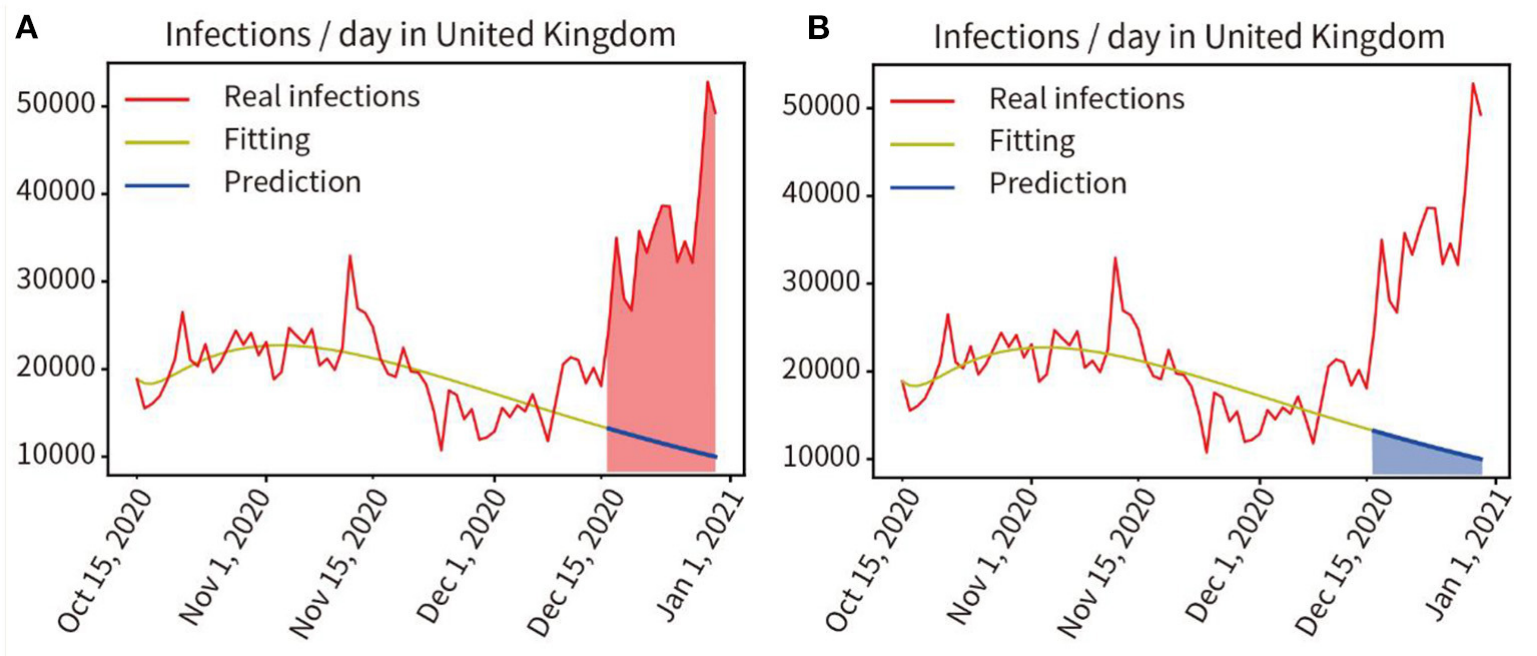
Quantitative analysis of the impact of virus mutation on the pandemic in the UK. **(A)** Red area indicates the integral under the curve of the actual infected individuals in the prediction period. **(B)** Blue area indicates the integral under the curve of the predicted infected persons in the prediction period.

**Figure 8:**
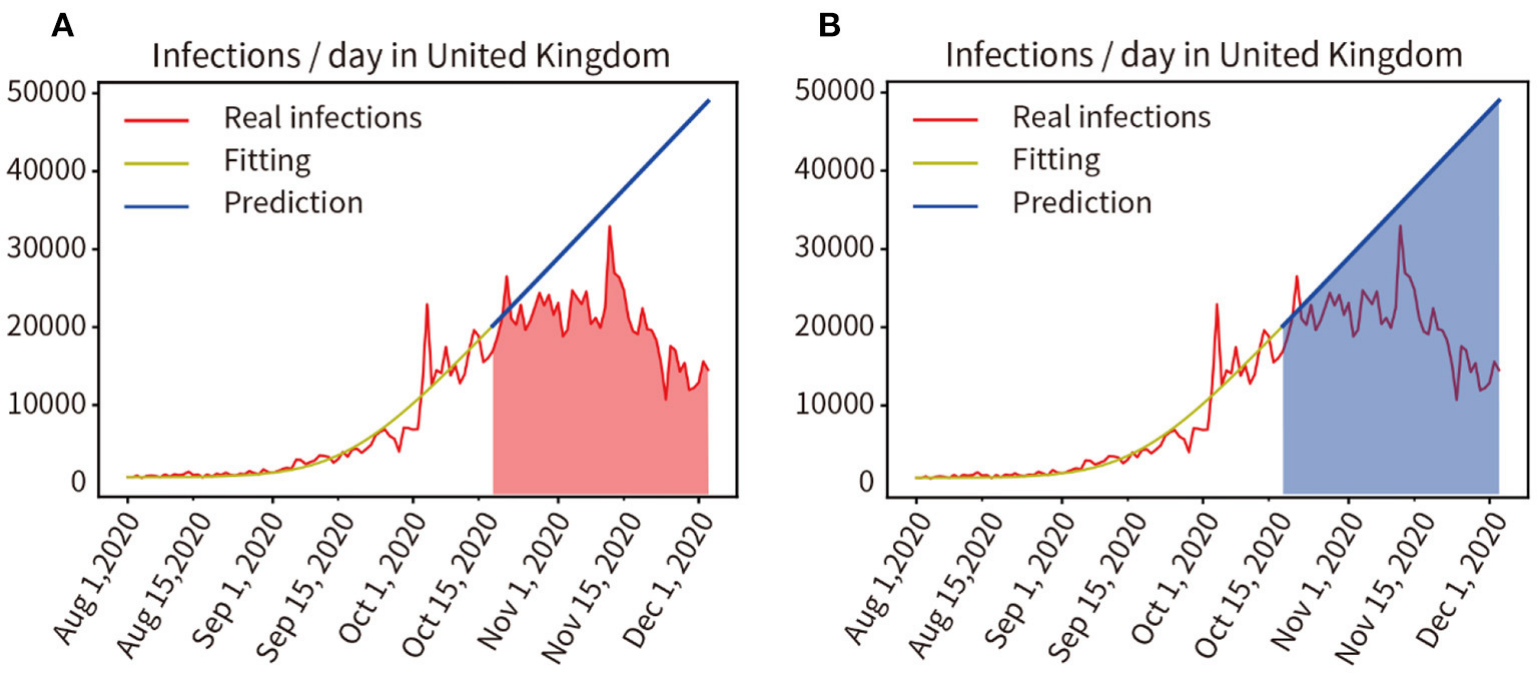
**(A,B)** Quantitative analysis of the impact of the three-tier social blockade on the pandemic in the UK.

In the UK, on 15 December 2020, a new coronavirus mutation B.1.1.7 was detected (21). We trained and fitted the model based on the pandemic data from 16 October to 15 December 2020, the fitting accuracy was 99.45%. The predicted infections from 16 December to 28 December, 2020 were performed without considering virus mutations, and the prediction curve was shown as the blue line in **Figure 8**. Here, we quantified the increment of total disease duration of infection due to viral mutation, and compared the predicted number of infections under the assumption of no viral mutation with the actual number of infections under the condition of mutant virus, comparing the curve integral of the real infections (Red area in Figure 7A) and that of the predicted infections (Blue area in Figure 7B), showing that the mutant virus caused a 301.55% increase in the number of infections (539,916:179,046 = 301.55%).

### Impact of the Three-Tier Social Blockade on the Pandemic in the UK

In August 2020, an outbreak of the COVID-19 pandemic escalated rapidly in the UK. A three-tier social lockdown was officially launched on October 16, 2020 (22). Training and fitting the pandemic data that did not adopt the three-tier social blockade from 1 August to 15 October, and the training and fitting results are shown in the yellow curve in Figure 8A, the accuracy of the fitting is 96.57%. Then, the progress of the pandemic was forecast from 16 October to 3 December in 2020 without considering the three-level lockdown, as shown by the blue line in Figure 8B. We compared the actual number of infected people from 16 October to 3 December in 2020 with the predicted number of infected people without the three-tier social lockdown, as indicated by the red and blue area in Figures 8A,B, the result was 985,137:1,859,053 = 52.99% (Real infections' integral: Predicted infections' integral). As a result, infections were reduced by 47.01% during the lockdown.

### Prospective Prediction of the Pandemic in Six Countries for the Next 15 and 30 Days

**Figure 9:**
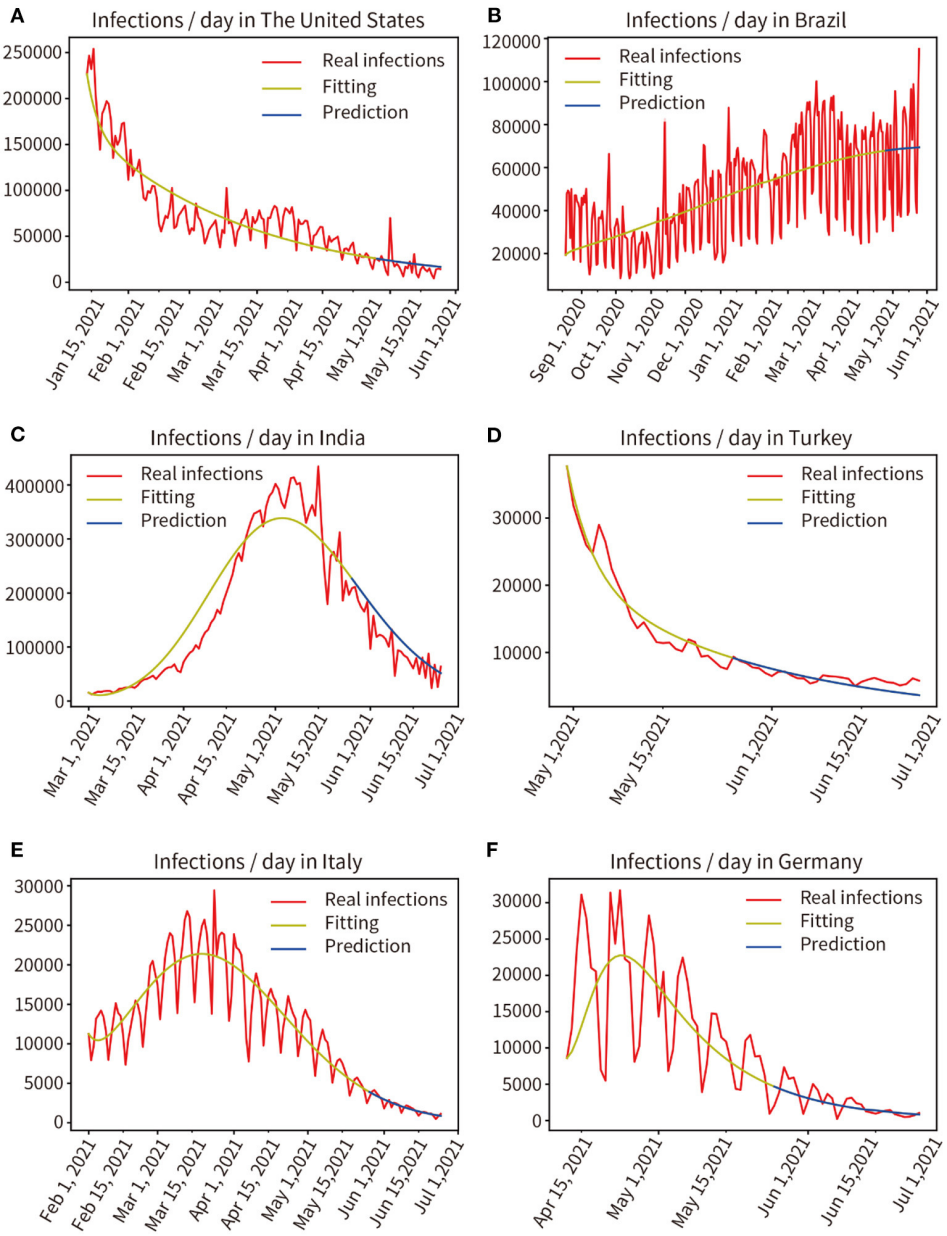
Real infected individuals are represented by the red curve. Fitted infections are represented by yellow curves. Predicted infections are shown as blue curves. **(A)** The United States, **(B)** Brazil, **(C)** India, **(D)** Turkey, **(E)** Italy, **(F)** Germany.

We modeled six countries severely affected by the pandemic based on real historical data up to 25 May 2021, generating forward projections for the next 30 days (26 May, 2021 to 24 June, 2021). This work was completed on 30 May 2021, the results are shown in Figure 9. Comparing the prediction results of the next 30 days with the real data, through mathematical integration, the accuracy of the previous fitting and the accuracy of the subsequent prediction are obtained, respectively, The United States (97.96, 83.77%), Brazil (98.20, 89.96%), India (96.17, 81.80%), Turkey (98.06, 92.30%), Italy (99.86, 93.17%), Germany (93.33, 88.89%). We selected the six countries mentioned above to be at the peak of a severe outbreak for prediction, and their subsequent control measures including vaccination, therefore deviated from the prediction, but the model showed a correct trend. Moreover, the prediction accuracy of the short-term 15-day pandemic progression was >30 days.

## Discussion

Capturing the complexity of factors accurately in the real world is difficult for any analytical model. Based on public datasets, we selected the data from 21 typical pandemic regions. Then, by modifying the classical SEIR transmission dynamics model and combining it with improved mathematical methods, compared with the traditional SEIR model, our newly added parameters “α' and “σ” enable the new model with the analysis function of vaccination and social isolation, and the newly added mathematical integral formula (10) under the curve realizes the quantitative analysis function. We conducted experiments in seven groups. By analyzing the effects of two vaccination methods, virus variation, and social lockdown, it is shown that the proposed method is capable of quantitatively analyzing and predicting the impact of different factors on a chaotic, multifactorial, and dynamically changing pandemic. This implies that the model can help the government develop a sound response strategy.

The experiments describe the dynamic transmission of COVID-19, provide evidence of the influence of various factors on the pandemic, highlight the adaptive capacity of the proposed model in different geographical and multi-factor situations, and the importance of interventions. According to the results of the present work, it is appropriate to select data from the period 60–150 days prior to the time point of analysis, this is critical for obtaining parameters that are consistent with the characteristics of the pandemic. The experience was used for the model to fit pandemic data from 13 regions distributed in different parts of the world, and the fitting accuracy reached 97.91%, showing that the model fitted the data well and a set of parameters adaptive to each region were obtained. Then, the pandemic trend for the next 30 days in each of these 13 regions was predicted by these parameters, and prediction accuracy is 87.53%, as shown in Figure 3 and Table 1. It shows that the model has good robustness, adaptability, and predictive power. Second, the effectiveness of vaccination against the pandemic under each of the two scenarios was quantitatively analyzed, as shown in Figures 4–6. It has been proved that in the absence of widely available therapies able to eliminate the infectious disease, the most important means to overcome COVID-19 is universal vaccination. We also found that the proportion of infected people continues to decrease over time following vaccination campaigns. Third, as shown in Figure 7, in the short period from 16 December 2020 to 14 January 2021, the variant virus caused a 301.55% increase in infections, which confirms the enormous danger of mutated viruses. Fourth, as shown in Figure 8, the number of infections in the United Kingdom was reduced by 47.01% owing to three-tier social distancing campaigns. Hence, social lockdown is essential in areas where vaccines are lacking. Fifth, the model was used to predict the pandemic course in six countries over the next 30 and 15 days, as shown in Figure 9. The results confirmed the accuracy of the model's predictions again. Also, the experiments suggest that, with the exception of the lack of a widespread vaccination campaign in Brazil, assuming that there are no more infectious virus variants, it seems unlikely that these countries will experience larger waves of pandemic outbreaks in the near future.

The proposed model does involve some limitations. First, the essential function of the new model and analysis method proposed in this paper is to quantitatively analyze the impact of various factors on the pandemic based on historical data. However, prediction of the future trend of the pandemic may sometimes be imprecise, and it is impossible to predict the pandemic for a very long time because interventions and virus mutations are dynamically changing. Second, the warehouse-based SEIR model requires the initial number of infections to be at least 15 people per day, and is suitable for the middle of the pandemic. Third, the setting of model parameters and the data used may be flawed; some of the model parameters have been set empirically, for example, the ratio of latent infections to fully infected people in the model was initially set at 1:3, which may not be accurate. Some of the actual pandemic data may not be available owing to opaqueness and incompleteness, for example, some asymptomatic infections may go undetected (31).

Despite the limitations of the new model, this study has made the following contributions: (1) A new SEIR model and a new analysis method using mathematical integral equations are proposed. (2) Only a few critical dynamic transmission parameters were used to avoid overfitting in model training. (3) The model's adaptability to different regions was verified experimentally; based on the historical pandemic data in a particular region, the model required only a few seconds of training and fitting to obtain the COVID-19 dynamic parameters of spread in a given region. (4) Several new analytical functions have been developed based on the compatibility and extensibility of the model considering impacts of vaccination, viral mutation, and social blockade on the course of the pandemic. (5) The robustness and accuracy of the model were validated through retrospective and prospective multi-center experiments. The proposed approach is expected to inform the development of interventions to mitigate, suppress, and control COVID-19.

## Conclusion

In this multi-center study, which included both retrospective and prospective modeling, we have shown the dynamic transmission process of COVID-19, the impact of different factors on the pandemic, and the prediction of future pandemic trends. We have focused on a quantitative analysis of the impact of two vaccination regimens, a variant mutant strain of the virus and social distancing requirements on COVID-19. The experimental results indicate that the integration of various interventions is required to suppress the pandemic. Moreover, the accuracy of short-term prediction is higher than that of long-term prediction. This quantitative analysis can inform the development of sound intervention strategies to balance economic losses and reduce human impacts. Owing to the adaptive nature of the model, the method can be used for various stages of the pandemic in different regions around the world. Although the proposed model has some limitations, the method constitutes a breakthrough in quantitatively analyzing the influence of chaotic and dynamic multi-factors on pandemic progression. Owing to the importance of the global public health emergency caused by the spread of COVID-19, we believe that the proposed new adaptive multi-factor multifunctional model and the analytical approach have certain applications.

## Data Availability Statement

The datasets presented in this study can be found in online repositories. The names of the repository/repositories and accession number(s) can be found in the article/supplementary material.

## Ethics Statement

As all the data of human participants were de-identied, the requirement for written informed consent and ethical approval was waived. All procedures performed in studies involving human participants were in accordance with the National Research Committee and the 1964 Helsinki declaration and its later amendments or comparable ethical standards. Written informed consent from the participants' legal guardian/next of kin was not required to participate in this study in accordance with the national legislation and the institutional requirements.

## Author Contributions

YP: project design, data analysis and proofreading, coding programming, experiment, visualization, and writing—original draft and editing. JL: data searching and proofreading. SX: proposing the research direction. YX: supervision and guidance. All authors read and approved the manuscript.

## Funding

This study was supported by a grant from Science and Technology Planning Project of Guangdong Province (2020B1111170001). The funding did not play any roles in the experimental design, data collection, analysis and interpretation, and manuscript writing.

## Conflict of Interest

The authors declare that the research was conducted in the absence of any commercial or financial relationships that could be construed as a potential conflict of interest.

## Publisher's Note

All claims expressed in this article are solely those of the authors and do not necessarily represent those of their affiliated organizations, or those of the publisher, the editors and the reviewers. Any product that may be evaluated in this article, or claim that may be made by its manufacturer, is not guaranteed or endorsed by the publisher.

## References

[1] CSSE. COVID-19 Dashboard by the Center for Systems Science and Engineering (CSSE) at Johns Hopkins University (JHU). CSSE (2021). Available online at: https://coronavirus.jhu.edu/map.html (accessed April 2, 2021).

[2] Smith LE Potts HWW Amlôt R Fear NT Michie S Rubin GJ. Adherence to the test, trace, and isolate system in the UK: results from 37 nationally representative surveys. BMJ. (2021) 372:n608. 10.1136/bmj.n608 33789843 PMC8010268

[3] Hadjidemetriou GM Sasidharan M Kouyialis G Parlikad AK. The impact of government measures and human mobility trend on COVID-19 related deaths in the UK. Transp Res Interdiscip Perspect. (2020) 6:100167. 10.1016/j.trip.2020.100167 34173458 PMC7334915

[4] WHO. Coronavirus Disease (COVID-19) Pandemic. WHO (2021). Available online at: https://www.who.int/ (accessed April 2, 2021).

[5] GOV.UK. Optimising the COVID-19 Vaccination Programme for Maximum Short-Term Impact. GOV.UK (2021). Available online at: https://www.gov.uk/government/publications/prioritising-the-first-covid-19-vaccine-dose-jcvi-statement/optimising-the-covid-19-vaccination-programme-for-maximum-short-term-impact (accessed February 27, 2021).

[6] Smith A. COVID-19 Vaccines Are Being Hoarded by Rich Countries — Poor Ones Are Missing Out. (2020). Available at: https://www.nbcnews.com/news/world/COVID-19-vaccines-are-being-hoarded-rich-countries-poor-ones-n1251351 (accessed April 2, 2021).

[7] Korber B Fischer WM Gnanakaran S Yoon H Theiler J Abfalterer W. Tracking changes in SARS-CoV-2 spike: evidence that D614G increases infectivity of the COVID-19 virus. Cell. (2020) 182:812–27.e19. 10.1016/j.cell.2020.06.043 32697968 PMC7332439

[8] Lemieux JE Siddle KJ Shaw BM Loreth C Schaffner SF Gladden-Young A. Phylogenetic analysis of SARS-CoV-2 in Boston highlights the impact of superspreading events. Science. (2021) 371:eabe3261. 10.1126/science.abe3261 33303686 PMC7857412

[9] Della Rossa F Salzano D Di Meglio A De Lellis F Coraggio M Calabrese C. A network model of Italy shows that intermittent regional strategies can alleviate the COVID-19 epidemic. Nat Commun. (2020) 11:5106. 10.1038/s41467-020-18827-5 33037190 PMC7547104

[10] Gu T Wang L Xie N Meng X Li Z Postlethwaite A. Toward a country-based prediction model of COVID-19 infections and deaths between disease apex and end: evidence from countries with contained numbers of COVID-19. Front Med. (2021) 8:585115. 10.3389/fmed.2021.585115 34179029 PMC8222531

[11] Reno C Lenzi J Navarra A Barelli E Gori D Lanza A. Forecasting COVID-19-associated hospitalizations under different levels of social distancing in lombardy and emilia-romagna, northern Italy: results from an extended seir compartmental model. J Clin Med. (2020) 9:1492. 10.3390/jcm9051492 32429121 PMC7290384

[12] Kissler SM Tedijanto C Goldstein E Grad YH Lipsitch M. Projecting the transmission dynamics of SARS-CoV-2 through the postpandemic period. Science. (2020) 368:860–8. 10.1126/science.abb5793 32291278 PMC7164482

[13] Russell TW Wu JT Clifford S Edmunds WJ Kucharski AJ Jit M. Effect of internationally imported cases on internal spread of COVID-19: a mathematical modelling study. Lancet Public Health. (2021) 6:e12–20. 10.1016/S2468-2667(20)30263-2 33301722 PMC7801817

[14] Bayham J Fenichel EP. Impact of school closures for COVID-19 on the US health-care workforce and net mortality: a modelling study. Lancet Public Health. (2020) 5:e271–8. 10.1016/S2468-2667(20)30082-7 32251626 PMC7270508

[15] Li M Zu J Li Z Shen M Li Y Ji F. How to Reduce the transmission risk of COVID-19 more effectively in New York city: an age-structured model study. Front Med. (2021) 8:641205. 10.3389/fmed.2021.641205 34485318 PMC8414980

[16] Saad-Roy CM Wagner CE Baker RE Morris SE Farrar J Graham AL. Immune life history, vaccination, and the dynamics of SARS-CoV-2 over the next 5 years. Science. (2020) 370:811–8. 10.1126/science.abd7343 32958581 PMC7857410

[17] World Bank. COVID-19 (Coronavirus) Response. World Bank (2020). Available online at: https://www.worldbank.org/en/topic/health/coronavirus (accessed April 2, 2021).

[18] New York State COVID-19 Vaccine Tracker. COVID-19 Vaccination Progress to Date. New York State COVID-19 Vaccine Tracker (2021). Available online at: https://covid19vaccine.health.ny.gov/covid-19-vaccine-tracker

[19] Hodgson SH Mansatta K Mallett G Harris V Emary KR Pollard AJ. What defines an efficacious COVID-19 vaccine? A review of the challenges assessing the clinical efficacy of vaccines against SARS-CoV-2. Lancet Infect Dis. (2021) 21:e26–35. 10.1016/S1473-3099(20)30773-8 33125914 PMC7837315

[20] Baden LR El Sahly HM Essink B Kotloff K Frey S Novak R. Efficacy and safety of the mRNA-1273 SARS-CoV-2 vaccine. N Engl J Med. (2021) 384:403–16. 10.1056/NEJMoa2035389 33378609 PMC7787219

[21] Kelland K. New Coronavirus Strain Spreading in UK has Key Mutations, Scientists Say. (2020). Available online at: https://www.reuters.com/article/health-coronavirus-britain-variant-idUSKBN28P158 (accessed April 2, 2021).

[22] Pylas P. England to Have 3-Tiger Lockdown System Amid ‘Tipping Point3-Tier Lockdown Point. (2020). Available online at: https://apnews.com/article/virus-outbreak-pandemics-england-london-europe-b2417cb4e92f7f832a3df2e944d1adf8 (accessed April 2, 2021).

[23] Korobeinikov A. Lyapunov functions and global properties for SEIR and SEIS epidemic models. Math Med Biol. (2004) 21:75–83. 10.1093/imammb/21.2.75 15228100

[24] Ashcroft P Huisman JS Lehtinen S Bouman JA Althaus CL Regoes RR. COVID-19 infectivity profile correction. Swiss Med Wkly. (2020) 150:w20336. 10.4414/smw.2020.20336 32757177

[25] Verelst F Willem L Beutels P. Behavioural change models for infectious disease transmission: a systematic review (2010–2015). J R Soc Interface. (2016) 13:20160820. 10.1098/rsif.2016.0820 28003528 PMC5221530

[26] Annas S Pratama MI Rifandi M Sanusi W Side S. Stability analysis and numerical simulation of SEIR model for pandemic COVID-19 spread in Indonesia. Chaos Solitons Fractals. (2020) 139:110072. 10.1016/j.chaos.2020.110072 32834616 PMC7345386

[27] van Dorp L Richard D Tan CC Shaw LP Acman M Balloux F. No evidence for increased transmissibility from recurrent mutations in SARS-CoV-2. Nat Commun. (2020) 11:5986. 10.1038/s41467-020-19818-2 33239633 PMC7688939

[28] Bury TM Bauch CT Anand M. Charting pathways to climate change mitigation in a coupled socio-climate model. PLoS Comput Biol. (2019) 15:e1007000. 10.1371/journal.pcbi.1007000 31170149 PMC6553685

[29] Dhar V. Data science and prediction. Commun ACM. (2013) 56:64–73. 10.1145/2500499

[30] Jordan MI Mitchell TM. Machine learning: trends, perspectives, and prospects. Science. (2015) 349:255–60. 10.1126/science.aaa8415 26185243

[31] Buitrago-Garcia D Egli-Gany D Counotte MJ Hossmann S Imeri H Ipekci AM. Occurrence and transmission potential of asymptomatic and presymptomatic SARS-CoV-2 infections: a living systematic review and meta-analysis. PLoS Med. (2020) 17:e1003346. 10.1371/journal.pmed.1003346 32960881 PMC7508369

